# Evidence of an increased pathogenic footprint in the lingual microbiome of untreated HIV infected patients

**DOI:** 10.1186/1471-2180-12-153

**Published:** 2012-07-28

**Authors:** Angeline T Dang, Sean Cotton, Sumathi Sankaran-Walters, Chin-Shang Li, Chia-Yuan Michael Lee, Satya Dandekar, Bruce J Paster, Michael D George

**Affiliations:** 1 Dept. of Medical Microbiology and Immunology, University of California, Davis, Davis, CA, USA; 2 Dept. of Molecular Genetics, Forsyth Institute, Cambridge, MA, USA; 3 Dept. of Public Health Services, University of California, Davis, Davis, CA, USA; 4 Dept. of Microbiology, Immunology, and Molecular Genetics, University of California, Los Angeles, Los Angeles, CA, USA; 5 Neuroscience Undergraduate Interdepartmental Program, University of California, Los Angeles, Los Angeles, CA, USA; 6 Dept. of Oral Medicine, Infection and Immunity, Harvard School of Dental Medicine, Boston, MA, USA

**Keywords:** HIV, Microbiota, Oral mucosa, Dysbiosis, CD4+ T cells, Streptococcus, Commensal, Microbiome, HOMIM

## Abstract

**Background:**

Opportunistic oral infections can be found in over 80% of HIV + patients, often causing debilitating lesions that also contribute to deterioration in nutritional health. Although appreciation for the role that the microbiota is likely to play in the initiation and/or enhancement of oral infections has grown considerably in recent years, little is known about the impact of HIV infection on host-microbe interactions within the oral cavity. In the current study, we characterize modulations in the bacterial composition of the lingual microbiome in patients with treated and untreated HIV infection. Bacterial species profiles were elucidated by microarray assay and compared between untreated HIV infected patients, HIV infected patients receiving antiretroviral therapy, and healthy HIV negative controls. The relationship between clinical parameters (viral burden and CD4+ T cell depletion) and the loss or gain of bacterial species was evaluated in each HIV patient group.

**Results:**

In untreated HIV infection, elevated viremia was associated with significantly higher proportions of potentially pathogenic *Veillonella*, *Prevotella*, *Megasphaera*, and *Campylobacter* species in the lingual microbiome than observed in healthy controls. The upsurge in the prevalence of potential pathogens was juxtaposed by diminished representation of commensal *Streptococcus* and *Veillonella* species. Colonization of *Neisseria flavescens* was lower in the lingual microbiome of HIV infected patients receiving antiretroviral therapy than in uninfected controls.

**Conclusions:**

Our findings provide novel insights into the potential impact of HIV infection and antiretroviral therapy on the community structure of the oral microbiome, and implicate potential mechanisms that may increase the capacity of non-commensal species to gain a stronger foothold.

## Background

Human immunodeficiency virus (HIV) infection leads to a progressive loss of CD4+ T cell numbers and function, impairing immune responses and rendering the host susceptible to secondary opportunistic infections [1-3]. Opportunistic infections (OI) of the oral mucosa are presented in up to 80% of HIV-infected patients [4], often causing debilitating lesions that contribute to deterioration in nutritional health. While, several studies have examined the effects of HIV infection on oral mucosal immunity in patients with OI [5,6], questions regarding the role of epithelial pathogenesis remain to be answered. Although the underlying mechanisms remain unknown, the oral epithelium appears to be more permeable and perturbed during HIV infection [7]. Studies in the simian immunodeficiency virus (SIV) non-human primate model may provide some mechanistic clues. Similar to the intestinal mucosa [8,9], SIV infection leads to a rapid down regulation of genes that mediate oral epithelial regeneration [10]. In addition to increasing barrier permeability, impairment of epithelial regenerative capacity is likely to enhance susceptibility to OI by disrupting homeostatic interactions with the overlying protective microbiota (microbiome).

The human oral microbiome is a complex polymicrobial community in delicate balance. The microbiota consists of a variety of aerobic and anaerobic species that produce a milieu of peptides and polysaccharides that interact with each other and with host molecules to maintain a stable symbiotic microenvironment [11-13]. More than 700 bacterial species have been detected in the human oral cavity, of which 35% are, so far, uncultivable [14]. In healthy oral tissues, access to the epithelium is vigorously protected from non-commensal organisms, due in part to the physical and physiological barriers supplied by the microbiome [15]. Microbial antigens such as lipopolysaccharide, flagellin, peptidoglycan, and fimbrae presumably contribute to this process as well. These antigens differentially stimulate innate response mechanisms through pattern recognition receptors (PRRs) and thereby regulate the local physiological environment. In turn, the physiological constraints dictate the corresponding profile of organisms the epithelial surface can support [16,17]. Although appreciation for the putative role that the microbiome can play in the initiation and/or enhancement of oral disease has grown considerably in recent years, little is known about the impact of HIV infection on host-microbe interactions within the oral cavity.

In the present study we provide, to our knowledge, the first characterization of modulations in the dorsal tongue (lingual) microbiota that are associated with chronic HIV infection. Lingual bacterial species were identified in oral swab samples utilizing the Human Oral Microbe Identification Microarray, or HOMIM (http://mim.forsyth.org/). Bacterial species profiles were compared between untreated chronically HIV infected patients, chronically HIV infected patients receiving antiretroviral therapy (ART), and healthy uninfected age matched controls. CD4+ T cell depletion and viral burden were measured in peripheral blood by flow cytometry and Amplicor viral load assays, respectively. Our findings provide novel insights into the impact of HIV infection on host-microbe homeostasis within the lingual microbiome, and reveal a potential correlation between high viremia and colonization of several putative opportunistic pathogens in untreated patients.

## Results

### HIV infected patients and healthy controls harbor similar quantities of lingual bacteria

To characterize alterations in the oral microbiome associated with chronic HIV infection and administration of antiretroviral therapy (ART), resident bacterial species profiles on the dorsal tongue epithelium were compared between 12 HIV infected patients (6 ART naïve, 6 receiving ART) and 9 healthy HIV-negative controls. The dorsal tongue surface was chosen for microbiome sampling because that anatomical site typically displays less sample to sample variation in microbial community structure compared to other oral niches, and because it is a common location for manifestation of HIV associated oral disease (e.g. candidiasis). One of the 6 HIV infected subjects on ART (#166) had a previous case of thrush, diagnosed 2–3 weeks prior to collection of the oral swab sample, but was not symptomatic or undergoing antibiotic treatment at the time of sample collection. None of the other treated or untreated HIV infected patients in the study had been previously or concurrently diagnosed with candidiasis or any other HIV associated oral complications. In addition, none of the HIV infected or control subjects were under concurrent antibiotic or antimycotic treatment during the study.

**Figure 1:**
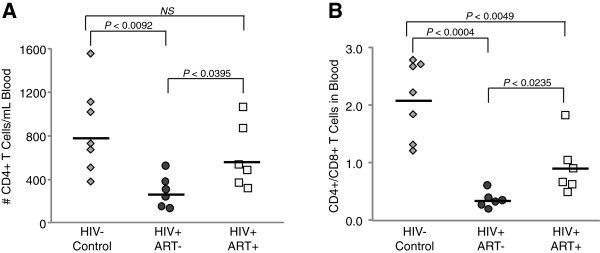
**Comparison of circulating CD4+ and CD8+ T cell subsets.** The absolute quantity of CD4+ T cells (**A**), and the ratio of CD4+ and CD8+ T cells (**B**) as reported in CBC analysis of the blood of HIV- controls, untreated HIV infected patients, and HIV infected patients receiving ART.

Patients in the ART naïve group had been HIV positive for at least one year, and displayed peripheral viral loads ranging from 9 x 10^3^ to 2 x 10^4^ RNA copies/mL blood and CD4+ T cells counts ranging from 525 to 137 cells/mL blood (Table 1). All HIV patients in the treated group had been receiving ART uninterrupted for at least 3 years, showed undetectable peripheral viral loads, and had CD4+ T cell counts in that ranged from 322 to 1069 cells/mL blood. Peripheral CD4+ T cell depletion was statistically significant in the untreated HIV infected group when compared to uninfected healthy controls (Figure 1A). HIV patients receiving long-term ART showed significantly higher CD4+ T cell numbers than untreated patients, although not reaching the levels observed in healthy controls. CD4/CD8 ratios in untreated HIV patients and HIV patients on ART were both significantly below the levels observed in healthy controls (Figure 1B).

**Table 1:** Study Participants

	**Patient ID**							**Time**		
		**Gender**	**Status**	**ART**	CDA*	CDA*	**VL+**	**HIV+**	**Time Tx**	**Ag69e**
HIV Control (n = 9)	204	F	Control	N/A	730	327	N/A	N/A	N/A	54
	206	F	Control	N/A	510	275	N/A	N/A	N/A	69
	213	F	Control	N/A	1021	382	N/A	N/A	N/A	43
	214	F	Control	N/A	1559	1294	N/A	N/A	N/A	36
	215	M	Control	N/A	380	290	N/A	N/A	N/A	57
	218	M	Control	N/A	674	241	N/A	N/A	N/A	35
	222	F	Control	N/A	ND	ND	N/A	N/A	N/A	24
	225	F	Control	N/A	ND	ND	N/A	N/A	N/A	55
	226	F	Control	N/A	1114	401	N/A	N/A	N/A	61
HIV ART-(n = 6)	217	M	HIV+	No	307	1117	216000	9 yr	None	39
	224	F	HIV+	No	238	1072	90000	3 yr	None	39
	207	F	HIV+	No	151	385	55000	1.5 yr	None	59
	221	M	HIV+	No	381	1188	50000	9 yr	None	41
	223	F	HIV+	No	137	389	18000	1 yr	None	30
	212	M	HIV+	No	525	873	9000	1.5y	None	28
HIV + ART + (n = 6)	158	M	HIV+	Yes	873	1302	UD	23 yr	20 yr	58
	166	M	HIV+	Yes	540	1041	UD	13 yr	13 yr	53
	205	F	HIV+	Yes	1069	582	UD	15 yr	8 yr	61
	208	F	HIV+	Yes	322	311	UD	6 yr	3 yr	37
	219	F	HIV+	Yes	490	531	UD	6 yr	6 yr	44
	228	M	HIV+	Yes	368	582	UD	9 yr	9 yr	39

Clinical characteristics of study participants. * CD4+, CD8+ T cells/mL blood. ^†^ HIV RNA copies/mL blood. ART = antiretroviral therapy, Tx = treatment.

**Figure 2:**
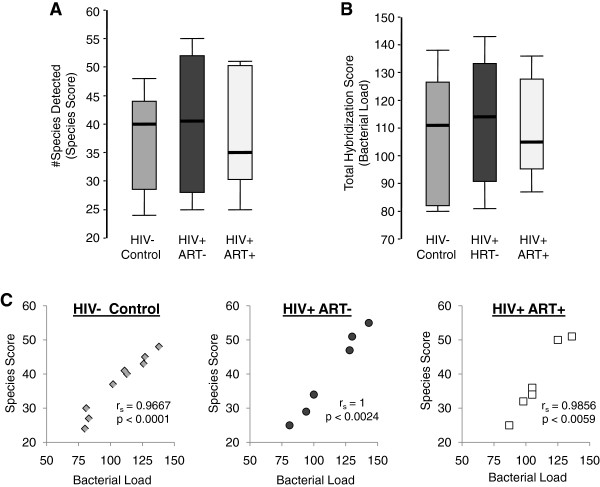
**HOMIM-based analysis of bacterial growth in the lingual microbiome.** (**A**) Comparison of the number of bacterial species (Species Score) detected by HOMIM assay on the tongue epithelium of healthy HIV- controls, ART naive chronically HIV infected patients, and HIV infected patients on ART. Median values are shown in horizontal bars. (**B**). HOMIM-based comparison of total bacterial populations (Bacterial Load) on the tongue epithelium of HIV- controls and HIV + patient groups. (**C**) Correlation between Species Score and Bacterial Load data as determined by Spearman rank correlation coefficient analysis.

To evaluate the general impact of chronic HIV infection and ART administration on the oral microbiota, we utilized the HOMIM to identify the number of bacterial species residing on the dorsal tongue surface of HIV infected patients and compared the species profiles to healthy controls. We first compared the mean number of bacterial species (all probes with signals ≥1) detected on the tongue between each patient and control group (Species Score) and found no significant differences (Figure 2A). To determine if there were differences in the total number of bacteria on the tongue (Bacterial Load), the total integer score for each sample was then tallied over all the probes on the array and mean values were compared between controls and HIV infected groups. Similar to the Species Score, no statistically significant difference was detected in Bacterial Load between uninfected and infected groups (Figure 2B). In addition, we found that Species Score and Bacterial Load data were highly correlated in individual samples across all experimental groups and controls (Figure 2C). Although the Species Score and Bacterial Load data does not address proportional shifts in bacterial species between experimental groups and controls, the findings do indicate that the capacity of the lingual epithelium to support complex polymicrobial communities was not impaired by chronic HIV infection or the administration of ART.

### Modulations in the lingual microbiome of HIV infected patients

**Figure 3:**
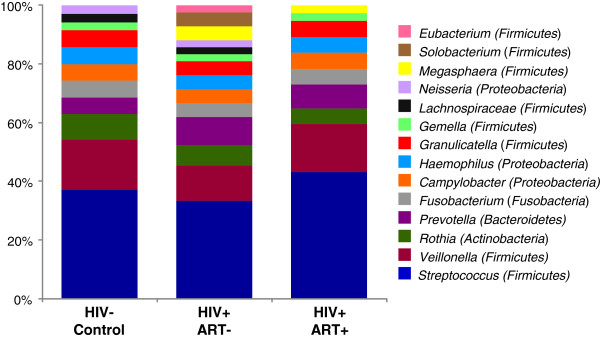
**Proportional taxonomic assignments at the genus level in controls and HIV + patient groups.** The relative proportions of the genera detected in the total lingual bacterial community in a majority of healthy controls, untreated HIV infected patients, and HIV patients on ART are represented by the height of their individual bars in the stacked bar graphs. Untreated HIV patients displayed an overall increase in genus representation, while HIV patients on ART showed a modest reduction.

To evaluate whether HIV infection was associated with alterations in the community structure of the lingual microbiota in HIV patients, we next analyzed the phylogenetic distribution of species that were detected in the majority of subjects in each patient group (Figure 3). As observed in previous studies, *Streptococcus* species dominated the oral microbiome of healthy subjects [18-21], comprising ~38% of all species detected by HOMIM, followed by *Veillonella* (~19% of all species) and *Rothia* (~7% of all species). In total, 11 different genera were represented in the oral microbiome of at least one-half of all healthy controls. In contrast, 14 genera were detected in ART naive HIV infected patients, which included all of the genera detected in healthy controls as well as *MegasphaeraEubacterium*, and *Solobacterium*. Notably, higher representation of these 3 genera appeared to be counterbalanced by lower relative proportions of core commensal *Streptococcus* and *Veillonella* species. Thus, the taxonomic findings also supported our observations that the “Bacterial Load” and “Species Score” generated by HOMIM analysis did not differ significantly between treated or untreated HIV infected groups and healthy controls.

Recent studies suggest that long-term ART may have adverse effects on the oral health of HIV infected patients [22]. In comparison to controls and untreated HIV patients, only 10 genera were represented in the oral microbiome of HIV patients undergoing ART. Representation from *Lachnospiraceae* and *Neisseria* was largely lost, while similar to the untreated HIV + group, *Megasphaera* colonization was higher than observed in healthy subjects. While not reaching statistical significance, the loss in prevalence of *Neisseria flavescens* was most striking, colonizing the microbiome of 67% of uninfected controls and untreated HIV patients, but only 17% (one subject) of HIV patients on ART. These data may be notable in light of reports that have linked reduced oral colonization by *N. flavescens* with increased incident of caries [23]. In agreement with Bacterial Load findings (Figure 2B), the lower relative proportions of *Lachnospiraceae* and *Neisseria* observed in the microbiome of HIV patients on ART appeared to be counterbalanced by higher relative proportions of other genera. In addition to *Megasphaera*, HIV patients on ART showed substantially higher colonization of *Streptococcus* species when compared to healthy controls and the ART naïve HIV + group. Collectively, these findings indicate that administration of ART may lead to alterations in the phylogenetic profile of the oral microbiota that are fundamentally distinct from the changes associated with untreated HIV infection.

### Association between HIV burden and colonization by potential opportunistic pathogens

**Figure 4:**
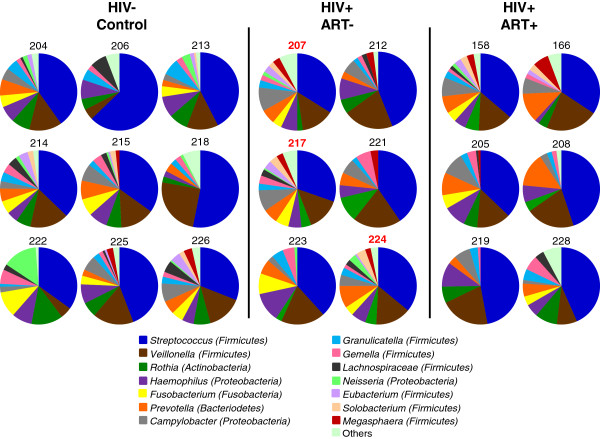
**Proportions of taxonomic assignments at the genus level in individual control subjects and HIV + patients.** The relative proportions of the genera detected in the total lingual bacterial community of each study participant are represented in pie charts. Similar genus distribution profiles were identified in 3 untreated HIV infected patients (207, 217, and 224: labelled in red text).

**Figure 5:**
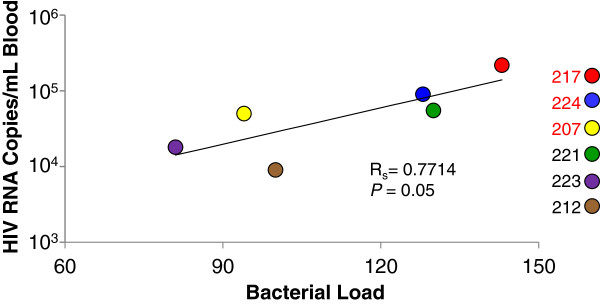
**Relationship between HIV burden and increased bacterial growth in the oral microbiome.** The relationship between viral loads in peripheral blood and the gain of bacterial growth (Bacterial Load score identified by HOMIM analysis) in ART naïve HIV infected patients was determined by Spearman rank correlation coefficient analysis. HIV infected patients that showed similar oral microbiome profiles are labelled in red text.

When the phylogenetic distribution of oral bacteria was evaluated in each patient individually, a substantial amount of variability within the experimental groups and controls was revealed (Figure 4). However, despite this variability, the phylogenetic profiles of 3 of the untreated HIV infected patients (207, 217, and 224 – labelled in red text) were strikingly similar. Further examination revealed that these 3 patients also displayed the highest levels of viral burden in our study cohort, and that each of the patients had <350 CD4+ T cells/mL of blood. Correlative analyses were then performed to evaluate the potential relationship between clinical parameters (viral replication and CD4+ T cell depletion) and modulations in the oral microbiome (Bacterial Load and Species Score data). Although data from additional samples will be needed to validate the trend, we observed a potential correlation between peripheral viral loads and Bacterial Load in ART naive HIV infected patients (Figure 5). A similar potential correlation was also observed between viral loads and Species Score (data not shown). Depletion of CD4+ T cells in the untreated HIV + group showed a similar but weaker trend towards correlation with Bacterial Load and Species Score. However, as with viral loads, high standard deviations associated with relatively small sample sizes prevented us from definitively linking CD4+ T cell depletion with differences in the oral microbiota between untreated HIV patients and healthy controls.

**Figure 6:**
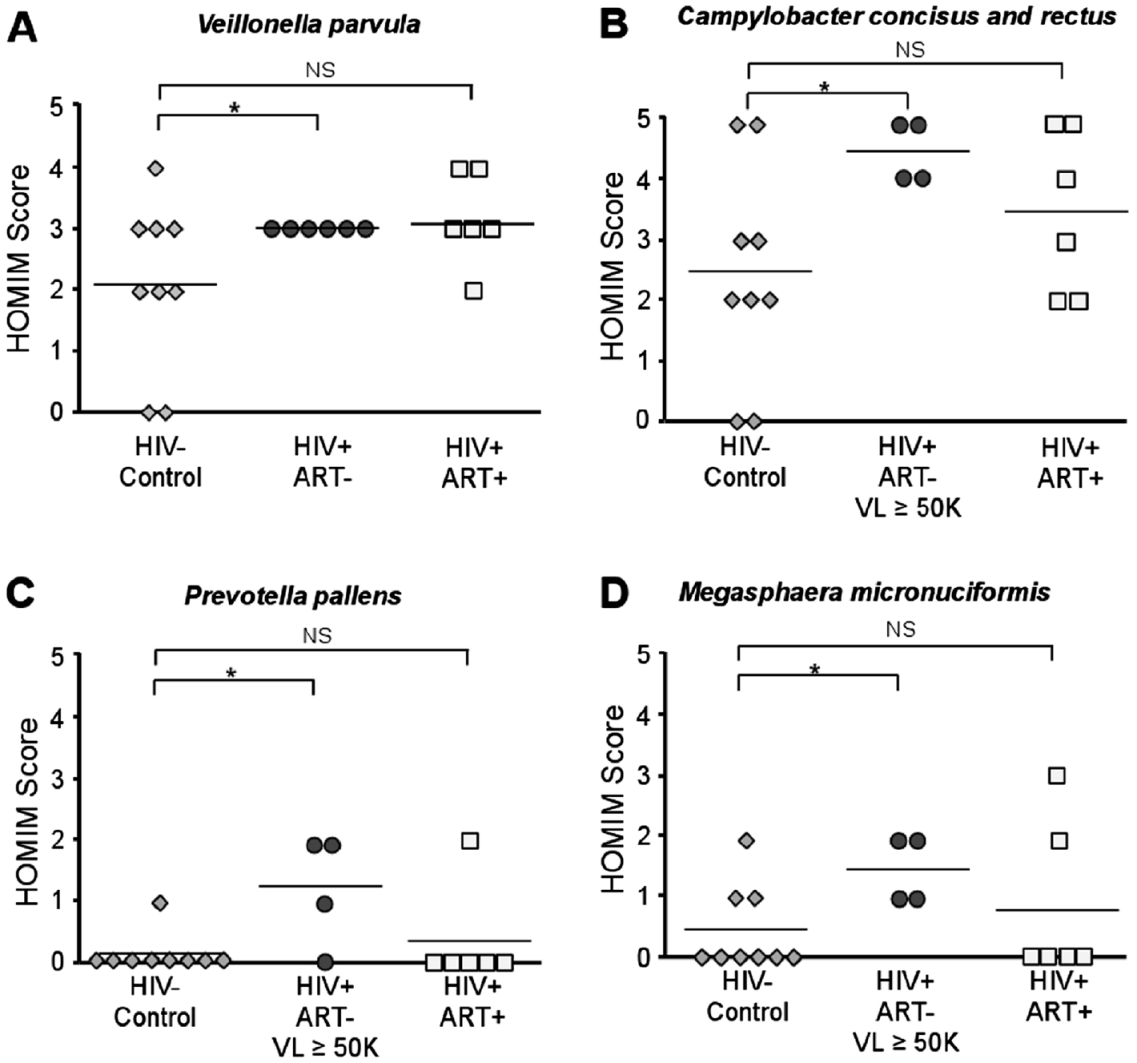
**Emergence of opportunistic pathogens in the oral microbiome of ART naive HIV infected patients.** (**A**) A statistically significant increase in the growth of *Veillonella parvula* was detected amongst all untreated HIV + subjects, while growth of (**B**) *Campylobacter concisus/rectus*, (**C**) *Prevotella pallens*, and (**D**) *Megasphaera micronuciformis* was significantly increased in untreated patients with HIV loads ≥ 50 K/mL of blood. Statistical analysis was performed using Wilcoxon rank-sum tests.

We next analyzed differences in the prevalence of individual bacterial species between untreated HIV infected patients and healthy controls. Although differences in the abundance of several species approached statistical significance when comparing the untreated HIV infected group as a whole to controls, these differences often became significant when comparing HIV infected patients with high viral loads (HVL). We defined HVL, for the purposes of our study, as viral burden ≥50 K HIV copies/mL blood. *Veillonella parvula* was the lone exception, displaying a significant difference in abundance (*P* = 0.042) from uninfected controls across the entire untreated HIV infected group (Figure 6A). We detected significant differences between HVL HIV patients and uninfected controls in the prevalence of *Campylobacter concisus* and/or *Campylobacter rectus* [cross-hybridizing HOMIM probe] (*P* = 0.032), *Prevotella pallens* (*P* = 0.027), and *Megasphaera micronuciformis* (*P* = 0.031) (Figures 6B-6D). Interestingly, most of the species displaying higher prevalence in HVL HIV patients have also been linked to periodontal pathogenesis, and *M. micronuciformis* has been identified in previous studies through its association with serious clinical infections [24]. Taken together, the presence of potential bacterial pathogens at higher abundance and commensal *Streptococcus* and *Veillonella* species at lower abundance than healthy controls indicates that uncontrolled HIV infection may be associated with a fundamental shift in the character of host-microbe interactions within the oral microbiome.

## Discussion

Maintenance of oral health is dependent on preserving the homeostatic balance between host and the distinct microbial communities that colonize the various anatomical microenvironments in the oral cavity. HIV infected patients often display increased susceptibility to opportunistic oral infections that are presumably linked, in part, to disruption of host-microbe homeostasis (dysbiosis). In the current study, we utilize HOMIM-based analyses to characterize and compare the bacterial composition of the lingual microbiome in a relatively small, but well-defined cohort of untreated chronically HIV infected patients (n = 6), HIV patients on ART (n = 6), and uninfected controls (n = 9). Due to the small sample sizes, it is important to caution that our findings represent a preliminary indication of the impact of HIV infection on the community structure of the oral microbiome. Indeed, the microbiome of even a single individual can be difficult to define, consisting of entrenched endogenous species and transient species whose prevalence can vary depending on time of sampling, diet, oral hygiene, and numerous other parameters [19]. Extensive cross sectional and longitudinal sampling of patients with and without oral manifestations will ultimately be necessary to fully characterize the role of the microbiota in HIV associated oral pathogenesis. The current study represents an important first step towards that goal. Our findings indicate that chronic HIV infection may lead to substantial disruptions in the community structure of the lingual microbiota, even in the absence of clinical oral manifestations.

Several potential mechanisms that have been revealed in previous studies may contribute to the development of host-microbe dysbiosis in the oral mucosa during immunodeficiency virus infection. Recently, analysis of SIV infected rhesus macaques demonstrated that, similar to the gut mucosa, depletion of CD4+ T cells from the oral mucosa is rapid and dramatic [10]. This finding underscores the likelihood that immune dysfunction resulting from the loss of CD4+ T cell activity in the oral cavity could contribute to the development of oral manifestations during SIV/HIV infection. Recent studies suggest that Notch-1 signaling mediates epithelial barrier function in the gut through interaction with CD4+ T cells [25]. Although interaction of Notch-1 with CD4+ T cells has not been studied in the oral mucosa, Notch-1 signaling is known to mediate oral epithelial cell differentiation [26]. Thus, it is possible that CD4+ T cell depletion from the oral mucosa of HIV infected subjects may also lead to the impairment of epithelial growth and, by extension, host-microbe dysbiosis.

In addition, depletion of the Th17 subset of CD4+ T cells has been shown in the gut mucosa impair response to microbial infections [8,27], in part by dampening expression of epithelial antimicrobial peptides [28]. HIV patients display decreased expression of histatin-5, a potent antimycotic known to inhibit the growth of *Candida albicans*[29]. Moreover, *in vitro* studies suggest that X4-tropic HIV can inhibit expression of human beta defensin-2 (hBD-2) and other innate immune factors in differentiated oral epithelium [30]. Because hBD-2 functions as a chemoattractant for dendritic cells in addition to its antimicrobial activity [31], the loss of hBD-2 during HIV infection could potentiate the colonization of pathogenic species through multiple mechanisms. Thus, it is conceivable that, similar to the gut mucosa, Th17 cells may be depleted from the oral mucosa in SIV/HIV infection, thereby providing a potential mechanism for increased susceptibility to dysbiosis and infection from *C. albicans* and other non-commensal pathogens.

Interestingly, one of the largest and most consistent alterations we detected in the oral microbiome of untreated HIV patients was a shift in the representation of *Veillonella* species. Although the relative percentage of *Veillonella* dropped from ~19% of the total lingual bacterial population in healthy controls to just over 10% in untreated HIV infected subjects, that same group displayed a uniform increase in the growth of *V. parvula*. While *V. parvula* is a commensal gram negative anaerobic coccus in healthy individuals [32], it is also the only known *Veillonella* species associated with oral disease. *V. parvula* has been implicated in severe early childhood caries [33], primary endodontic infections [34], and other periodontal diseases [35]. Recent studies indicate that *V. parvula* lipopolysaccharide (LPS) stimulates pro-inflammatory cytokine production and p38 MAPK activation through TLR-4 dependent mechanisms [36]. Thus, it is possible that increased *V. parvula* colonization (as well as other opportunistic pathogens) could establish an inflammatory environment in the oral cavity, that in turn, contributes to the chronic inflammation and immune activation that characterizes HIV disease progression. Future studies are warranted to determine whether increased colonization of putative periodontal pathogens on the tongue epithelium reflects similar increased growth in gingival and subgingival tissues, and perhaps a systemic distribution to more distal mucosal compartments.

## Conclusions

In summary, we identify statistically significant increases in the growth of *V. parvulaP. pallensC. rectus* and/or *C. concisus*, and *M. micronuciformis* in the lingual microbiome of untreated HIV infected subjects, albeit a small patient cohort where previous oral infections could not be considered. Due to advances in therapeutic efficacy and clinical care in developed countries, susceptibility of HIV patients to opportunistic oral infections has been dramatically reduced [37,38]. However, worldwide, where the vast majority of HIV infected individuals do not have access to basic clinical care or therapy, oral complications remain a serious problem [39,40]. Large-scale sampling from an appropriate range of geographic and cultural regions and collation of data from multiple studies will lead to a more complete understanding of host-microbe dysbiosis in HIV infection. To that end, the HOMIM and similar high throughput methodologies designed for rapid identification of microbial profiles may represent ideal cost-effective tools for accomplishing such ambitious large-scale endeavors.

## Methods

### Patients and sample collection

All participants were enrolled through the Center for AIDS Research, Education and Services (CARES) clinic in Sacramento, CA after providing informed written consent. The research was carried out according to Institutional Review Board (IRB)-approved procedures (219139–5) and in compliance with the Helsinki Declaration. The oral health status of each patient was determined prior to participation in the study, including any recent or concurrent periodontal procedures and history of candidiasis and other oral infections. Patients undergoing antibiotic or antimycotic treatment were excluded from the study. Pertinent clinical data was also obtained on all participants. These data included duration of HIV infection, CBC with differential, CD4+/CD8+ T cell numbers (blood was not collected from 2 of the 9 uninfected control subjects), peripheral blood HIV viral loads, and duration of antiretroviral therapy. Peripheral blood viral load assays were performed at the CARES clinical lab using the Amplicor HIV-1 Assay (Roche Molecular Diagnostics). Two-sided Satterthwaite’s and Student’s t-tests were utilized to determine the statistical significance of differences in T cell subsets between uninfected controls and HIV infected patient groups.

During the same clinical appointment that blood samples were obtained, tongue epithelial samples were collected utilizing non-invasive swabbing of the dorsal surface. Briefly, MasterAmp Buccal Swabs© (Epicentre Biotechnologies, Inc) were used to collect epithelial cells and resident microbes, and DNA was extracted utilizing the protocols and reagents provided in the Epicentre MasterAmp© kit. Extracted DNA was transferred into new tubes and stored at −20°C until HOMIM analysis**.**

### HOMIM processing

Identification of oral bacterial species and quantitation of their relative proportions was carried out using the Human Oral Microbe Identification Microarray, or HOMIM [41]. Briefly, 16 S rRNA genes (rDNAs) were PCR amplified utilizing a universal primer set (forward primer, 5'-GAG AGT TTG ATY MTG GCT CAG; reverse primer, 5'- GAA GGA GGT GWT CCA RCC GCA), and Cy3-dCTP-labeled. Purified, labelled 16 S amplicons were then hybridized to the printed HOMIM slides at 55°C for 16 h. Hybridized slides were washed and dried and Cy3 fluorescence was detected using the GenePix 4000B microarray scanner (Axon) with photomultiplier settings (PMT) of 650 and wavelength of 532 nm.

### Analysis of HOMIM data

Analysis of HOMIM data was performed as previously described [42,43]. Briefly, hybridization spot intensities were converted to one of the 6 integer signal levels ranging from 0 to 5, with 0 representing undetectable (above background) and 5 being the maximal intensity among all the profiles being compared. The number of bacterial species (Species Score) present in each sample was determined by summation of all probes with detectable signal (integer score ≥ 1), and a qualitative representation of the total bacteria (Bacterial Load) in each sample was estimated by summation of all integer scores. Correlations between “Species Score” and “Bacterial Load” were analyzed using Spearman rank correlation coefficient. Correlations between clinical parameters (viral loads, CD4+ T cell counts) and a gain or loss of oral bacteria were identified by Spearman rank correlation coefficient analysis. Wilcoxon rank-sum tests were utilized to determine if increases or decreases in individual bacterial species in HIV patient groups were statistically significant compared to healthy HIV- controls. The HOMIM data utilized in the study has been deposited in the Gene Expression Omnibus microarray database (Accession#: GSE38908).

## Competing interests

The author(s) declare that they have no competing interests.

## Authors' contributions

ATD collected samples, extracted DNA for HOMIM analysis, and drafted the manuscript. SC performed HOMIM assays. SS recruited patients for the study and collected samples. CL participated in the design of the study and performed statistical analyses. CML performed statistical analyses. SD participated in the design of the study and edited the manuscript. BJP participated in the design and coordination of the study and edited the manuscript. MDG conceived of the study and its design, directed its coordination, and helped to draft the manuscript. All authors read and approved the final manuscript.
